# Exercise Induces Peripheral Muscle But Not Cardiac Adaptations After Stroke: A Randomized Controlled Pilot Trial

**DOI:** 10.1016/j.apmr.2015.12.018

**Published:** 2016-04

**Authors:** Sarah A. Moore, Djordje G. Jakovljevic, Gary A. Ford, Lynn Rochester, Michael I. Trenell

**Affiliations:** a Institute of Cellular Medicine, Newcastle University, Newcastle upon Tyne, United Kingdom; b Research Councils UK, Newcastle Centre for Ageing and Vitality, Newcastle upon Tyne, United Kingdom; c University of Oxford, Oxford, United Kingdom; d Institute of Neuroscience, Newcastle University, Newcastle upon Tyne, United Kingdom

**Keywords:** Cardiac output, Exercise, Physical fitness, Rehabilitation, Stroke, 6MWT, 6-minute walk test, 10MWT, 10-meter walk test, BBS, Berg Balance Scale, CRF, cardiorespiratory fitness, SLE, single limb exercise, TUG, timed Up and Go

## Abstract

**Objective:**

To explore the physiological factors affecting exercise-induced changes in peak oxygen consumption and function poststroke.

**Design:**

Single-center, single-blind, randomized controlled pilot trial.

**Setting:**

Community stroke services.

**Participants:**

Adults (N=40; age>50y; independent with/without stick) with stroke (diagnosed >6mo previously) were recruited from 117 eligible participants. Twenty participants were randomized to the intervention group and 20 to the control group. No dropouts or adverse events were reported.

**Interventions:**

Intervention group: 19-week (3times/wk) progressive mixed (aerobic/strength/balance/flexibility) community group exercise program. Control group: Matched duration home stretching program.

**Main Outcome Measures:**

(1) Pre- and postintervention: maximal cardiopulmonary exercise testing with noninvasive (bioreactance) cardiac output measurements; and (2) functional outcome measures: 6-minute walk test; timed Up and Go test, and Berg Balance Scale.

**Results:**

Exercise improved peak oxygen consumption (18±5 to 21±5mL/(kg⋅min); *P*<.01) and peak arterial-venous oxygen difference (9.2±2.7 to 11.4±2.9mL of O_2_/100mL of blood; *P*<.01), but did not alter cardiac output (17.2±4 to 17.7±4.2L/min; *P*=.44) or cardiac power output (4.8±1.3 to 5.0±1.35W; *P*=.45). A significant relation existed between change in peak oxygen consumption and change in peak arterial-venous oxygen difference (*r*=.507; *P*<.05), but not with cardiac output. Change in peak oxygen consumption did not strongly correlate with change in function.

**Conclusions:**

Exercise induced peripheral muscle, but not cardiac output, adaptations after stroke. Implications for stroke clinical care should be explored further in a broader cohort.

An audio podcast accompanies this article.

Listen at www.archives-pmr.org.

Cardiorespiratory fitness (CRF) levels are decreased after stroke,^1^ potentially leading to an increased risk of further cardiovascular disease.^2^ The criterion standard measure of CRF is peak oxygen consumption, which is the product of the capacity of the cardiovascular system to supply oxygen (ie, cardiac output) and the capacity of skeletal muscles to use oxygen (ie, arterial-venous oxygen difference). In healthy individuals, peak oxygen consumption appears to be limited by the cardiovascular system.^3^ The relation between peak oxygen consumption and oxygen supply and oxygen utilization has yet to be established poststroke. Two small cross-sectional studies have presented opposing views on the physiological basis of peak oxygen consumption poststroke. The first study^4^ indicated that reduced peak oxygen consumption is secondary to a decline in peak and reserve cardiac output. The second, more recent study,^5^ demonstrated that peak oxygen consumption and the ability of skeletal muscles to extract oxygen is reduced poststroke, but cardiac function and pumping capability are maintained.

Investigating how exercise mediates central oxygen supply and peripheral oxygen utilization may lead to a greater understanding of peak oxygen consumption poststroke and how it can be improved. Stroke can lead to a number of negative peripheral skeletal muscle adaptations (eg, change in muscle fiber type and vasculature).^6^ Central comorbidities such as heart disease and hypertension are also highly prevalent in this population.^7^ Exercise is a potential intervention capable of promoting both central and peripheral adaptations, and these changes may affect both function and CRF poststroke.

This study aims to explore central and peripheral adaptations to exercise poststroke and the physiological mechanisms that are related to exercise-induced changes in peak oxygen consumption and function. Our hypotheses are that after stroke, (1) structured exercise will improve central oxygen supply and peripheral oxygen utilization; (2) exercise-induced change in peak oxygen consumption will be strongly associated with adaptations in both central oxygen supply and peripheral oxygen utilization; and (3) exercise-induced change in peak oxygen consumption and peripheral muscle oxygen utilization will be strongly associated with improvements in function.

## Methods

### Study design

The study design was a single-center, single-blind, randomized controlled pilot trial. The trial was approved by the County Durham and Tees Valley Research and Ethics Committee. All participants gave informed written consent for the study. The study was performed in accordance with the ethical standards laid down in the 1975 Declaration of Helsinki and as revised in 2013. Primary outcomes for this study have been reported previously.^8^ This article presents a subanalysis of the primary findings.

### Participants

#### Eligibility criteria

The inclusion criteria for the study were as follows: (1) age >50 years; (2) stroke diagnosed (>6mo previously) by a stroke specialist; (3) able to complete a 6-minute walk test (6MWT) with or without a stick; (4) living at home; (5) discharged from all conventional physiotherapy interventions; and (6) not already performing regular exercise (≥3times/wk, moderate intensity). The exclusion criteria were as follows: (1) the absolute and relative contraindications to exercise testing as stated by the American Heart Association^9^; (2) diabetes; (3) neurological disorders other than stroke; (4) pain on walking (visual analog scale score, >5); (5) inability to follow 2 stage commands; (6) cognitive impairment (Mini-Mental Scale Examination score, <24); and (7) untreated major depression.

#### Setting

Participants were recruited from community stroke services by National Institute for Health North East Stroke Local Research Network clinical trial officers, stroke health professionals, or advert.

#### Exercise intervention

The intervention was adapted from the Fitness and Mobility Exercise Program designed by Eng in 2006.^10^ The intervention was a “mixed” exercise program consisting of functional exercises designed to improve flexibility, strength, aerobic capacity, and balance. The intervention was delivered using a previously described protocol.^8^ In brief, classes were delivered in the community for 19 weeks by a fitness instructor and a physiotherapist (3times/wk, 45–60min). Participants wore a heart rate monitor,a and the intensity of the exercise was gradually increased, working within a heart rate zone determined using the Karvonen formula^11^ (40%–50% of participant's maximum heart rate, with increasing increments of 10% every 4wk up to 70%–80%). Repetition and resistance were used to progress strength and balance exercises.

#### Control group intervention

The control group completed a matched duration home stretching program. Ten seated stretches were repeated 3 times for the upper and lower body. Participants were given an instruction booklet and diary to record activity and changes in medication/diet/physical activity and telephoned fortnightly for progress.

#### Outcomes

Primary outcomes have been published previously.^8^ Outcomes listed below represent only the variables explored in this secondary analysis: cardiorespiratory and functional performance measures. Outcome assessment was conducted within 2 weeks preintervention and 1 week postintervention by trained assessors blinded to the study hypotheses and group assignment.

#### Exercise testing

Expired gases (METALYZER 3Bb) were collected at rest for 5 minutes and continuously during a maximal progressive exercise test conducted with an electromagnetically controlled recumbent bicycle ergometer (Corivalc). A warm-up was done at 20W for 3 minutes followed by 10-W increments every minute until volitional exhaustion. The 12-lead electrocardiogram (Custod) was continuously monitored, and blood pressure was recorded twice at rest, during exercise, and at peak exercise and recovery. *Peak exercise* was defined as a respiratory exchange ratio of >1.05; the absence of an increase in oxygen consumption despite a further increase in exercise intensity; a rating of perceived exertion of >18 on the category Borg scale or voluntary termination of the test.^12^

#### Cardiorespiratory fitness

Peak oxygen consumption was calculated as the average oxygen uptake during the last minute of exercise (expressed in milliliters per kilogram per minute). *Peak work rate* was defined as the peak wattage on test termination.

#### Cardiovascular system

Cardiac hemodynamics were measured using a bioreactance system (NICOMe) following a previously described protocol.^13^ Bioreactance has been demonstrated to be a valid and reliable method for estimating cardiac output at rest and during the different stages of graded exercise testing, including maximal exertion.^13^, ^14^ Cardiac output was calculated as stroke volume × heart rate. Mean arterial blood pressure was calculated as diastolic blood pressure + (1/3) (systolic blood pressure – diastolic blood pressure) (expressed in millimeter of mercury), and cardiac power output was calculated as (cardiac output × mean arterial blood pressure) × (2.22×10^−3^).^5^

#### Peripheral muscle oxygen extraction

Peak arterial-venous oxygen difference (expressed in milliliters of oxygen per 100 milliliters of blood) was calculated as the ratio between peak oxygen consumption and peak cardiac output.

#### Functional outcome measures

The functional outcome measures were as follows: 6MWT,^15^ 10-meter walk test (10MWT),^16^ timed Up and Go (TUG) test,^17^ and Berg Balance Scale (BBS).^18^

#### Randomization

An allocation sequence to randomize to either the exercise or the control group was created using a computer “true” random number generator (www.random.org) and delivered after screening by an administrator not associated with the trial.

#### Statistical methods

There were no missing values in the data set; therefore, all participants were included in the analysis. Data were inspected for outliers using standard Z-distribution cutoffs and Mahalanobis distance tests. Normality of distribution was tested using a Kolmogorov-Smirnov test. Pre- and postintervention within-group analyses were performed using a paired *t* test if data conformed to the assumptions of normality; if not, then the Wilcoxon signed rank test was used. Between-group differences were assessed using change scores that were compared using independent sample *t* tests.

Pearson (or Spearman rank) correlation coefficient was used to explore associations between change scores in different variables. Cutoff scores to interpret effect size were .10 for small, .30 for moderate, and .50 for large.^19^ Statistical significance was indicated if *P*<.05. All data are presented as means ± SD or as otherwise indicated. The sample size calculation for the main trial was presented in our previous publication of the main trial findings.^8^

## Results

### Participant characteristics

**Fig 1:**
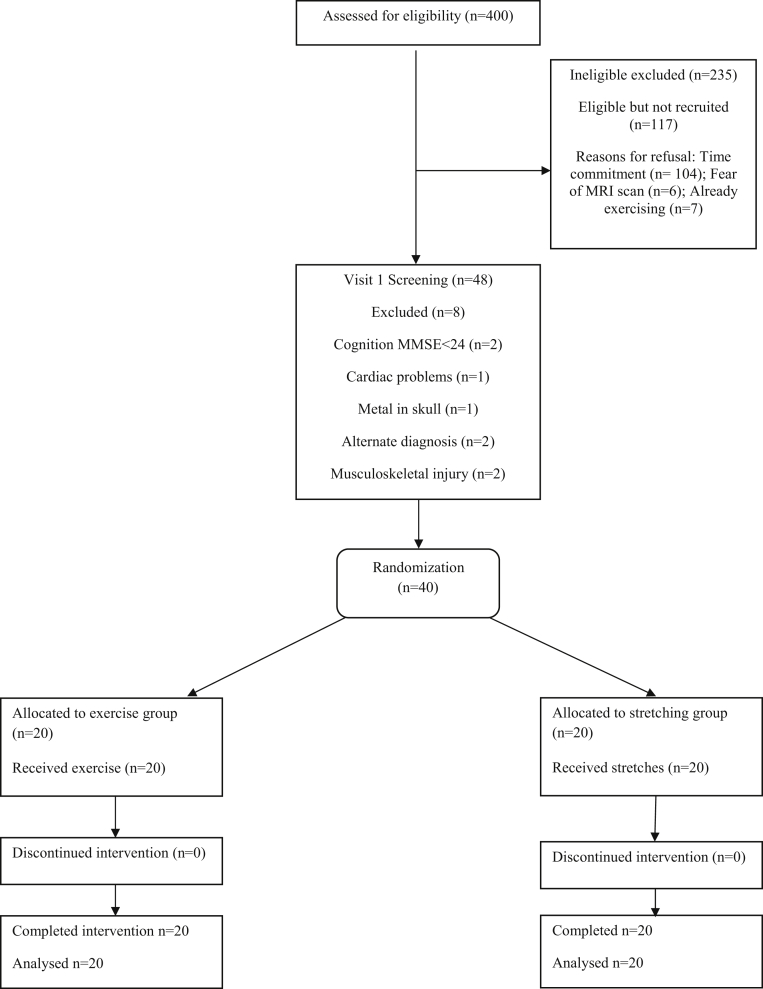
Flow diagram of study participants (CONsolidated Standards Of Reporting Trials diagram). Abbreviations: MMSE, Mini-Mental Scale Examination; MRI, magnetic resonance imaging.

A review of baseline characteristics and trial feasibility has been described previously.^8^ In brief, 400 patients were assessed to determine eligibility for the trial. Forty participants, with matching baseline characteristics, were randomized and completed the trial (see the CONsolidated Standards Of Reporting Trials diagram in fig 1). The trial sample size provided sufficient power to explore our primary outcome.^8^ The cohort included 34 men (85%) and 6 women (15%) (mean age, 69±9 years). Time since stroke was on average 19±26 months (range, 6–144mo). Impairment levels of participants were mild to moderate (National Institute for Health Stroke Scale score range, 0–8). All participants achieved the prespecified criteria for peak exercise. Normality testing was performed for all variables as described in the Methods section, and all but 3 data sets conformed to the assumptions of normality.

### Main results

#### Hypothesis 1: Exercise will improve central oxygen supply and peripheral oxygen utilization

Exercise significantly improved peripheral oxygen utilization as measured by peak arterial-venous oxygen difference. Exercise did not alter central oxygen supply as measured by baseline and peak cardiac output and cardiac power output (table 1).

**Table 1:** CRF and functional outcome measures at baseline and 19wk for exercise and control groups

Variable	Exercise Group (n=20)				Control Group (n=20)				Group × Time (95% Confidence Interval)
	Baseline	19wk	Δ	*P*	Baseline	19wk	Δ	*P*	
Cardiorespiratory measures									
Peak oxygen consumption (mL/(kg⋅min))	18±5	21±5	3	<.01∗	18±5	18±5	0	.62	<.01∗ (1.3 to 5.2)
Peak work rate (W)	112±36	121±37	9	<.01∗	105±34	101±35	−4	.13	<.01∗ (0.1 to 0.3)
Peak arterial-venous oxygen difference (mL of O_2_/100mL of blood)	9.2±2.7	11.4±2.9	2.2	<.01∗	9.1±2.4	10.1±3.0	1	<.05†	0.09 (−0.2 to 2.5)
Peak cardiac output (L/min)	17.2±4	17.7±4.2	0.5	.44	16.2±2.9	15.8± 2.6	−0.4	.26	0.23 (−0.5 to 2.1)
Peak cardiac power output (W)	4.8±1.3	5.0±1.35	0.2	.45	4.6±0.93	4.2±1.4	−0.5	.1	0.145 (−0.1 to 0.9)
Baseline cardiac output (L/min)	5.6±1.2	5.54±1.4	−0.1	.93	5.8±0.8	5.42±0.8	−0.4	.03†	0.12 (−0.1 to 0.9)
Baseline cardiac power output (W)	1.26±0.4	1.26±0.4	0	.91	1.29±0.2	1.25±0.2	−0.0	.36	0.6 (−0.1 to 0.18)
Physical performance									
6MWT distance (m)	428±131	513±131	85	<.01∗	419±127	441±126	22	<.05†	<.01∗ (42 to 86)
10MWT speed (m/s)	1.2±0.4	1.5±0.3	0.3	<.01∗	1.2±0.3	1.3±0.3	0.1	.01†	<.01∗ (0.1 to 0.3)
TUG test score (s)	11±9	8.4±6	−2.6	<.01∗	9.8±5	9±5	−0.8	.06	<.05† (−3.5 to −2.4)
BBS score	50±4	55±2	5	<.01∗	50±5.6	52±5	2	<.05†	<.01∗ (0.9 to 5)

NOTE. Values are mean ± SD or as otherwise indicated. Group x time indicates between group differences using change scores (significance and confidence intervals).

Abbreviation: Δ, change score.

∗ *P*<.01.

† *P*<.05.

#### Hypothesis 2: Exercise-induced change in peak oxygen consumption will be strongly associated with adaptations in both central oxygen supply and peripheral oxygen utilization

**Fig 2:**
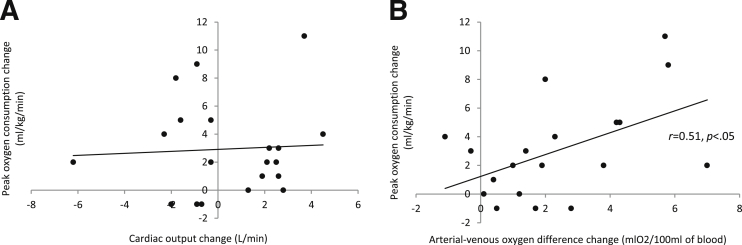
Relation (A) between peak oxygen consumption and cardiac output and (B) between peak oxygen consumption and arterial-venous oxygen difference.

Peak arterial-venous oxygen difference increased by 24% with exercise (see table 1). Change in peak oxygen consumption was significantly correlated with change in arterial-venous oxygen difference (*r*=.51; *P*<.05), but not significantly correlated with either peak cardiac output (*r*=.06; *P*=.82) (fig 2) or peak cardiac power output (*r*=.01; *P*=.98).

#### Hypothesis 3: Exercise-induced change in peak oxygen consumption and peripheral muscle oxygen utilization will be strongly associated with improvements in function

Significant between-group differences were demonstrated in favor of the exercise intervention in all functional outcome measures including 6MWT, 10MWT, TUG test, and BBS (see table 1). Exercise-induced changes in peak oxygen consumption and peripheral muscle oxygen utilization were not strongly associated with improvements in function (table 2). A moderate significant correlation was observed between exercise-induced change in peak oxygen consumption and change in 10MWT and TUG test scores (see table 2). No other significant correlations were demonstrated between changes in peak oxygen consumption and peripheral muscle oxygen utilization and changes in function.

**Table 2:** Correlations between change in peak oxygen consumption and arterial-venous oxygen difference and change in functional outcome measures

Functional Outcome Measure (Change Scores)	Peak Oxygen Consumption Change Score (mL/(kg⋅min))	Arterial-Venous Oxygen Difference Change Score (mL of O_2_/100mL of blood)
6MWT		
*r*	−.04	−.02
*P*	.88	.92
10MWT		
*r*	.48	.15
*P*	<.05∗	.53
TUG test		
*r*	.47	.04
*P*	<.05∗	.86
BBS		
*r*	−.25	−.4
*P*	.28	.12

Abbreviations: *P*, 2-tailed significance level; *r*, Pearson correlation.

∗ 2-tailed significance level.

## Discussion

This is the first study to explore the effect of exercise on cardiac and peripheral muscle adaptations poststroke. It is also the first study to explore physiological mechanisms associated with exercise-induced changes in peak oxygen consumption and function poststroke. Community-based exercise led to marked improvements in peak oxygen consumption and peripheral muscle oxygen utilization. Exercise did not alter central hemodynamics. Exercise-induced change in peak oxygen consumption was related to the ability of the skeletal muscles to extract oxygen, rather than cardiac function. Although exercise-induced improvements were demonstrated in both peak oxygen consumption and functional outcome measures, these improvements were not strongly related.

We have previously reported that the ability of skeletal muscles to extract oxygen is reduced poststroke, but cardiac function and pumping capability are not altered.^5^ Present findings extend this knowledge, demonstrating that structured exercise led to an improvement in peripheral muscle oxygen utilization, but did not alter cardiac function. These findings may reflect that the individuals studied had adequate cardiac output and cardiac power output at study baseline; therefore, there was no potential for improvement in contrast to the improvements observed in peripheral muscle oxygen utilization.

To our knowledge, only 1 other study^20^ has investigated the effect of exercise on cardiac function poststroke. Although cardiac hemodynamics were assessed using echocardiography in this study, not bioreactance as in our trial, the study used an exercise intervention of a similar frequency, duration, and intensity. To our own meaning, findings may be comparable. In line with our findings, most cardiac measures did not alter with exercise, with right atrial emptying fraction percentage being the only measure to alter.^20^ The study assessed cardiac function only at rest, whereas our study also analyzed cardiac function at peak exercise. Assessing cardiac function at peak exercise may be a more accurate representation of cardiac function as it takes into account cardiac adaptability to functional demand. In line with measures taken at rest, cardiac measures taken at peak exercise also did not alter with exercise, providing further novel evidence that cardiac hemodynamics may not be altered by exercise poststroke. However, in both studies participants were recruited at least 6 months poststroke. The participants may have initially presented with reduced cardiac function in the early stages of stroke, which may have been resolved by early physiotherapy and exercise interventions. To our knowledge, little research has been conducted on cardiac function and how it is affected by exercise in the early stages of poststroke and this area warrants further investigation.

Although no change was noted in cardiac hemodynamics in our study, exercise-induced change was observed in peripheral muscle oxygen utilization. Stroke can lead to peripheral impairments including severe unilateral muscle atrophy,^21^ an increase in intramuscular fat,^22^ a shift toward fast twitch, fatigue prone muscle fibers,^23^ and a marked increase in the expression of tumor necrosis factor α.^24^ Peripheral changes including vascular remodeling^25^ and a reduction in blood flow^26^ have also been observed poststroke in the femoral artery of the affected lower limb.

The exercise-induced improvements in peripheral muscle observed during this study indicate that it may be possible to use exercise to mediate peripheral complications resulting from stroke. The most effective form of exercise to promote these peripheral changes, however, has yet to be discovered. Our intervention combined functional strengthening, balance work, flexibility, and aerobic exercise. A more specific peripheral training regime, however, may have increased peripheral adaptation.

A 4-week (3times/wk) program of single limb exercise (SLE) has been shown to effectively modify peripheral hemodynamics.^27^ The SLE program increased femoral artery blood flow and diameter, which may have enhanced oxygen uptake in the hemiparetic limb. Although the SLE program led to vascular changes, the overall peak oxygen consumption was not altered, limiting the application of this intervention.^28^ Reasons for the lack of change in peak oxygen consumption may have been due to the relatively short training period used in the study and methods used for measuring CRF (exercise testing was performed on a total body recumbent stepper). A longer SLE training regime may lead to peripheral adaptations and greater improvements in peak oxygen consumption. An SLE program focusing on the hemiparetic limb, however, may not lead to the global improvements in CRF and function observed in response to the functional training program delivered in our study.^8^

Patient dissatisfaction after stroke is often related to functional outcome, rather than reduced CRF as measured by peak oxygen consumption.^29^ The exercise intervention delivered in this trial led to significant improvements in all the functional measures undertaken. Although previous data have demonstrated that peak oxygen consumption levels are associated with functional outcome measures in various conditions,^30^, ^31^ our results demonstrated only a moderate significant correlation between exercise-induced change in peak oxygen consumption and 2 of the functional outcome measures: walking speed and TUG test. No significant relations were observed between peak oxygen consumption and the other functional outcome measures (10MWT and BBS) and between peripheral muscle oxygen utilization and all the 4 functional outcome measures.

Previous cross-sectional studies^32^, ^33^ have demonstrated that after stroke, measures of CRF do not correlate strongly with functional outcome measures such as 6MWT. Our study supports these findings, demonstrating that exercise-related improvements in peak oxygen consumption were not strongly related to functional improvements as first hypothesized. This lack of relation between function and physiology could be due to impairments associated with stroke, such as balance, fatigue, cognition, and mood, affecting performance on functional tests that may not have affected performance on the exercise test conducted while sitting on a recumbent bike. Indeed, balance appears to be one of the strongest predictors of 6MWT distance,^32^ and 6MWT distance has also been previously correlated to quality-of-life measures.^34^

An interesting finding yet to be discussed was the improvement observed in the control group in peak arterial-venous oxygen difference and 3 of the functional outcome measures. There is some evidence to suggest that measures such as 6MWT may be subject to learning effects.^35^ Learning effects may have accounted for the functional improvements observed; however, maximal cycle ergometer testing has not been linked to the same type of effect.^36^ The intervention delivered to the control group was a low-intensity stretching program that was not designed to improve function or fitness, but may have led to the physiological and functional changes observed in the control group. However, the between-group analysis demonstrated that any improvements in the control group were significantly lower than those in the exercise group.

### Study limitations

The strength of this study lies in the novel exploration of exercise-induced peripheral and central adaptations poststroke. The study is, however, not without limitations. The trial did not allow for follow-up; therefore, we were not able to establish whether short-term changes were maintained. To be eligible to take part in the trial, participants had to be able to complete a 6MWT and they were from a single center, limiting the applicability of the study findings to a broader population with stroke. The cohort recruited was also predominantly male, with mild disability also reducing external validity. However, it is still important to explore responses in patients with nondisabling strokes as they are linked to further stroke, cardiovascular events, and death.^37^

## Conclusions

This study is the first to explore the effect of exercise on cardiac and peripheral muscle adaptations and the relation between exercise-induced changes in physiology and function. The exercise program led to significant improvements in peak oxygen consumption and peripheral muscle oxygen utilization, with no improvement in cardiac output and cardiac power output. Exercise-induced change in peak oxygen consumption was related to peripheral adaptations, but not to central adaptations or function. Future research should establish the most effective interventions to promote change in peripheral muscle oxygen utilization, peak oxygen consumption, and function poststroke.

## Suppliers

a. Heart rate monitor RS400; Polar, Finland.
b. METALYZER 3B; Cortex, Leipzig, Germany.
c. Corival; Lode, Groningen, The Netherlands.
d. Custo; CustoMed GmbH, Ottobrunn, Germany.
e. CHEETAH NICOM; Cheetah Medical, Vancouver.

## Supplementary data

Audio
